# Natural nasal–esophageal fiberscopy in the COVID-19 pandemic—preventing sneezing without anesthesia: a case report

**DOI:** 10.1186/s40001-021-00523-9

**Published:** 2021-06-09

**Authors:** Koichi Tsunoda, Ko Hentona, Yoshiharu Yamanobe

**Affiliations:** 1 Department of Artificial Organs and Medical Creations and Otolaryngology, National Hospital Organization Tokyo Medical Center, 2-5-1 Higashigaoka, Meguro-ku, Tokyo, 152-8902 Japan; 2 Department of Oto-Rhino-Laryngology, National Hospital Organization Tokyo Medical Center, Tokyo, Japan

**Keywords:** Natural phonatory functions, Natural swallowing function, Trans-nasal esophageal fiberscope, Safety technique, Sitting position esophageal observation without anesthesia, Glycogenic acanthosis, Tone enhancement scan (TE scan), Narrow band imaging (NBI)

## Abstract

**Background:**

We are laryngologists. We observe natural phonatory and swallowing functions in clinical examinations with a trans-nasal laryngeal fiberscope (TNLF). Before each observation, we use epinephrine to enlarge and smooth the common nasal meatus (bottom of nostril) and then insert a wet swab inside the nose, as in taking a swab culture in the nasopharynx. During the current COVID-19 pandemic situation, this careful technique prevents any complications, including nasal bleeding, painfulness, and induced sneezing. Here, we introduce our routine to observe esophageal movement in swallowing in a natural (sitting) position without anesthesia.

**Case presentation:**

The case was a 70-year-old female who complained that something was stuck in her esophagus; there was a strange sensation below the larynx and pharynx. After enlarging and smoothing the common nasal meatus, we inserted the TNLF (slim type ⌀2.9 mm fiberscope, VNL8-J10, PENTAX Medical, Tokyo, Japan.) in the normal way. We then observed the phonatory and swallowing movements of the vocal folds. As usual, to not interfere with natural movements, we used no anesthesia. We found no pathological condition in the pyriform sinus. We asked the patient to swallow the fiberscope. During the swallow, we pushed the TNLF and inserted the tip a bit deeper, which made the fiberscope easily enter the esophagus, like in the insertion of a nasogastric tube. We then asked the patient to swallow a sip of water or saliva to clear and enlarge the lumen of the esophagus. This made it possible to observe the esophagus easily without any air supply. With tone enhancement scan, the esophagus was found to be completely normal except for glycogenic acanthosis.

**Conclusions:**

The advantage of this examination is that it is easily able to perform without anesthesia and with the patient in sitting position. It is quick and minimally invasive, enabling observation the physiologically natural swallowing. It is also possible to observe without anesthesia down to the level of the esophagogastric junction using with a thin type flexible bronchoscope. In the future, gastric fiberscopes might be thinner, even with narrow band imaging (NBI) function. Before that time, physicians should remember to just insert along the bottom of the nose.

**Supplementary Information:**

The online version contains supplementary material available at 10.1186/s40001-021-00523-9.

## Background

We are laryngologists. We observe natural phonatory and swallowing functions in every clinical examination with a trans-nasal laryngeal fiberscope (TNLF). Before each observation, we use epinephrine to enlarge and smooth inside the common nasal meatus (bottom of nostril), and then insert a wet swab inside the nose, as in taking a swab culture in the nasopharynx (Additional file 1: Video S1). In cases of hypertension, we use a physiological saline solution to moisten inside the nose to insert the fiberscope smoothly and prevent any effect on phonation and articulation.

In COVID-19 pandemic situations, it is necessary to standardize acquisition of a nasopharyngeal culture to ensure accuracy of Polymerase Chain Reaction (PCR) studies [1]. For this reason, a revised technique was introduced [2]. To ensure satisfactory nasopharyngeal culture, we insert the swab along the nasal septum at the bottom of the nasal meatus below the inferior turbinate, with careful not to touch the inferior turbinate. It is important to never touch the inferior turbinate. We never anesthetize a patient’s nose when taking a nasopharyngeal culture. In this time of COVID-19, this careful technique prevents any complications, including nasal bleeding, painfulness, and induced sneezing [1]. Here, we introduce our routine safety technique to observe esophageal movement in swallowing in a natural sitting position without anesthesia.

## Case report

The case was a 70-year-old female who complained that something was stuck in her esophagus; there was a strange sensation below the larynx and pharynx. After enlarging and smoothing the common nasal meatus (same as shown in Additional file 1: Video S1), we inserted the trans-nasal laryngeal fiberscope (TNLF) (slim type ⌀2.9 mm fiberscope, VNL8-J10, PENTAX Medical, Tokyo, Japan.) in a similar way. (Additional file 2: Video S2, 0:00–0:03) Then, we observed the phonatory and swallowing movement of the vocal folds (0:06–0:16). To not interfere with natural movement, we did not use anesthesia. We found no pathological condition in the pyriform sinus. We asked the patient to swallow the fiberscope (0:18). During the swallow, we pushed the TNLF and inserted the tip a bit deeper which made the fiberscope easily enters the esophagus like the insertion of a nasogastric tube. When we then asked the patient to swallow a sip of water or saliva, the lumen of the esophagus cleared and enlarged (0:25). This makes it easy to observe the esophagus without any air supply. The esophagus was found to be completely normal (0:43) except for glycogenic acanthosis observed with a tone enhancement scan [3] (TE scan) (0:51–0:56).

## Discussion and conclusions

Physiological study of swallowing under topical anesthesia with lidocaine [4] has shown that sensory inputs from the mucosal receptors are important to trigger voluntary swallowing and their absence or dysfunction may contribute to oropharyngeal dysphagia and laryngeal aspiration. Another study based on 186 healthy volunteer study showed that there is an effect of different body postures on the self-perceived difficulty while swallowing. In comparison with all other tested postures, self-perceived difficulty for swallowing was found to be least, while subjects were sitting upright [5].

The advantage of this technique to observe physiologically natural swallowing is that it can be performed easily in natural sitting position without anesthesia, it takes only a minute and it is minimally invasive. It is also possible to observe without anesthesia down to the esophagogastric junction using with a thin type flexible bronchoscope. In the future, diameter of gastric fiberscopes might be gradually getting smaller, even with narrow band imaging (NBI) function [6] or TE scan [3]. Before that time, physicians should remember to just insert along the bottom of the nose.

## Supplementary Information

**Additional file 1: Video S1.** Swab culture in the nasopharynx: insert the swab along the nasal septum and bottom of nasal meatus below the inferior turbinate, attach along the bottom of septum and bottom of common nasal meatus.

**Additional file 2: Video S2.** Insert the trans-nasal laryngeal fiberscope to observe the esophagus.

## Data Availability

All data generated or analyzed during this study are included in this published article (and its Additional files).

## Abbreviations

TNLF: Trans-nasal laryngeal fiberscope
PCR: Polymerase chain reaction
TE: Tone enhancement scan
BI: Narrow band imaging
